# Factors Influencing Compliance with The Utilization of Effective Malaria Treatment and Preventive Measures in Wulu, South Sudan

**DOI:** 10.4314/ejhs.v30i4.5

**Published:** 2020-07-01

**Authors:** Gabriel O. Ayeni, Oladapo M. Olagbegi, Thayananthee Nadasan, Okwuoma C. Abanobi, Ebenezer O. Daniel

**Affiliations:** 1 Doctors with Africa CUAMM, Lakes State, South Sudan; 2 Department of Physiotherapy, School of Health Sciences, University of KwaZulu-Natal, Durban, South Africa; 3 Department of Public Health Technology, Federal University of Technology, Owerri, Imo State, Nigeria; 4 Department of Public Health School of Public Health, Texila American University Georgetown, Guyana, South America

**Keywords:** Compliance, effectiveness, malaria, prevention, treatment

## Abstract

**Background:**

High incidence of malaria disease in South Sudan may be largely due to poor/non-compliance with effective treatment and preventive measures. This study examined factors limiting/enhancing compliance with the utilization of known and effective malaria treatment and preventive measures in Wulu, South Sudan.

**Methods:**

A case-control study involving 396 respondents was conducted. Participants were interviewed using a semi-structured questionnaire to elicit information regarding socio-demographics and factors influencing compliance with using available treatment and preventive measures for malaria.

**Result:**

Respondents diagnosed with malaria reported lack of insecticide treated nets (51.5%) and forgetfulness (16.6%) as reasons for not using insecticide treated nets. About 26% of them lacked the knowledge of insecticide treated net's usefulness, while 57.5% of them did not consider it necessary to have door/window barriers. About 44% of all respondents forgot to take prescribed drugs at the right time while 14.5% of them did not complete drug prescriptions because they felt relief of symptoms. There were significant associations between identified factors of compliance to treatment/preventive measures and occurrence of malaria (all at p = 0.001). Having insecticide treated nets (OR: 5.78; CI: 3.46–9.00), awareness of its benefits (OR: 8.76; CI: 3.02–25.37), being taught on its use (OR: 3.35; CI: 2.17–5.18) and understanding of its use (OR: 3.80; CI: 2.01–7.20) were significantly associated with year-round utilization of insecticide treated nets.

**Conclusion:**

Poor access to and knowledge of malaria treatment, control and preventive measures are leading barriers to their effective utilization in Wulu.

## Introduction

Malaria remains a life-threatening disease caused by Plasmodium parasites among which *P. falciparum* and *P. vivax* species are the most prevalent and pose the greatest public health threat (1). The WHO’s 2017 report indicated that an estimated 219 million cases of malaria with associated 435000 deaths were reported in 87 countries (1). At the start of 2016, nearly half of the world’s population was at risk of malaria (WHO, 2016). Malaria was considered to be endemic in 91 countries and territories in 2016, down from 108 in 2000 (2). Most of the positive trend could be linked to the wide-scale deployment of malaria control interventions (2). However, the WHO’s summary of 2015-2017 malaria incidence and mortality data did not show significant progress as regards the global control of the disease during the period (3).

Unfortunately, the African continent bears a very high share of global malaria burden as the region accounted for 92% of malaria cases and 93% of malaria deaths in 2017 (1). The 2016 world malaria report showed that South Sudan was among countries that have not achieved more than 20% reduction in incidence and mortality between 2010 and 2015 (2). In spite of reported declining trend in the incidence of malaria diseases globally, the burden of the disease is still very high in South Sudan (3). According to the WHO’s World Malaria Report 2018 (3), the country reported over 2 million RDT confirmed cases of malaria with about 3500 deaths in 2017. The entire South Sudanese population is at risk of malaria which is still the leading cause of morbidity and mortality, accounting for 59% of cases and over 28% of deaths reported in health facilities in 2018 (4). Children under five years and pregnant mothers are the most vulnerable (4). Identifying possible factors for this should be of concern to public health authorities and stakeholders in the country.

Reduction in the burden of malaria is attributed to a scale-up of combinations of control strategies (5). In the main, these were long-lasting insecticide-treated nets (LLINs) or insecticide treated nets (ITNs), indoor residual spraying (IRS) and intermittent preventive therapy for pregnant women (IPTp) for prevention, better diagnostics for case ascertainment, and effective treatments using artemisinin-based combination therapies (ACTs) (5). The larger numbers of directly observed studies did not generally single out ITNs as the sole or major driver of the decline as recently suggested (6). In sub-Saharan Africa, the proportion of children under 5 years of age and sleeping under ITNs has increased from =2% in the year 2000 to an estimated 68% in 2015, although the estimates vary widely between countries (5). Despite these gains, inefficient distribution, ownership, and actual use of ITNs has potentially undermined its effectiveness (7). Investments in health systems and improved availability of ACTs and rapid diagnostic tests (RDTs) through global subsidies have also played a role (5). Other approaches are under investigation, and some have shown promise but not yet been widely deployed, including seasonal malaria chemoprevention (SMC) (8) and mass drug administration (MDA) (9). Furthermore, WHO recommends for malaria endemic areas, a package of prevention methods to reduce morbidity and mortality associated with the disease (5). Among pregnant women, the core preventive interventions are vector control through the provision and use of insecticide-treated bed nets (ITNs) and intermittent preventive treatment during pregnancy (IPTp) to prevent pregnancy-associated malaria (5). The combination of both prevention strategies has been found to be cost-effective and is associated with substantial reduction in neonatal mortality and low birth weight (10). The long-lasting insecticide treated net (LLIN) has been identified as a major preventive tool which, if well utilized, could reduce the burden of malaria (2,11). The utilization of ITNs has been shown to reduce malaria incidence rate by 50% and mortality rates by 55% in children under-5 years in sub-Saharan Africa (12). South Sudan through its national malaria control programme has also implemented some of these interventions, especially the LLIN and malaria treatment.

In the area of National policy and guidance, Southern Sudan’s MOH issued a health policy document in 2006 that emphasized the need for malaria control and the same ministry developed a strategic plan to cover malaria control between 2007 and 2013 which was also revised and Factors Influencing Compliance.

relaunched for the 2014–2021 period (13,14). The plan prioritized the distribution of LLINs. The initial target of the distribution (60% household coverage) was soon raised to 80% household coverage in subsequent guidelines (15). In 2007, the Global Fund to Fight AIDS, Tuberculosis and Malaria financed the scale-up of LLIN distribution in Southern Sudan, with the stated aim of reducing malaria incidence in the region by at least 50% by 2010 (13).

Wulu is a typical county in South Sudan with a population of about 60,000. More than 60% of the inhabitants are agrarian earning livelihood in crop production and animal husbandry. Others are artisan with few engaged in office or white-collar jobs. Data from the District Health Information System indicated that the area recorded a consistently high incidence of malaria during 2014-2017. This trend in the pattern of malaria occurrence in the area makes the county a good location for an investigation into factors promoting or limiting compliance with malaria preventive measures, In addition, the county shares socio-cultural and geographic similarities with other counties of South Sudan.

In Africa, malaria has a huge negative effect on economic growth and perpetuates a vicious cycle of poverty because it costs the continent above US $10 billion every year in lost gross domestic product (16). In spite of some progress made so far, the economic and morbidity burden as a result of malaria could still be said to be significant judged by the global incidence data (16). Promoting factors which enhance the uptake and or compliance with recommended treatment and preventive measure would tremendously help to reduce the health and economic burden of malaria. Furthermore, this is necessary to achieve WHO’s set target of reducing global malaria incidence and mortality rates by at least 90% by 2030 (5). There is, however, dearth of published data on factors limiting compliance to the utilization of treatment and preventive measures of malaria.

Despite considerable financial support given to South Sudan regarding scaling up LLIN coverage from the Global Fund to Fight AIDS, Tuberculosis and Malaria’s rounds 2, 7 and 10 for malaria control, and other funding agencies, it is noteworthy that malaria remained endemic in all 10 of South Sudan’s administrative states in 2012 (13). With malaria being accountable for 65% of all illnesses reported in health facilities across South Sudan (1), one could lend support to the possibility of low compliance with the utilization of the disease’s control measures. Therefore, the objective of this study was to investigate the challenges and factors that influence compliance with the utilization of known and effective malaria treatment and preventive measures in Wulu, South Sudan.

## Materials and Methods

### Research design

A case control design was adopted for the study.

### Setting

The study was conducted in Wulu County, Western Lakes State, South Sudan. The county is one of 4 major counties of Western Lakes State. The population of the county at the time of the study (2017) was estimated at 61,084 according to the District Health Information System (DHIS). Wulu County (named after one of the subcounty or payams) comprised 4 subcounties (geopolitical divisions) (Wulu, Makundi, Dulomoto and Bhargel) with Wulu town as the administrative seat of the county. The payams are further subdivided into 11 catchment areas, Wulu and Dulomoto Payams are made up of three catchment areas each, Bhargel has 4 catchment areas while Makundi has only one.

### Study population

The study population was male and female residents of Wulu County aged 16–60 years.

### Sampling size and sampling technique

With the assumption of a minimum odds ratio of 2 and power of 80%, an estimated minimum sample size of 272 (136 each for cases and controls) was calculated for the study using the formula for case control studies described by Charan and Biswas (17) and estimated proportion of control exposed (47%) reported by Chanda et al. (13).

A multistage sampling technique was used to select the study samples. Based on the population of each of the 4 payams, a systematic random sampling was used to select 847 households from the eleven catchment areas in the county. A simple random sampling was thereafter used to select a total of 396 respondents for the study. A higher number of respondents than the calculated sample size was surveyed to account for non-respondents. The respondents were confirmed cases of malaria and controls, malaria cases were confirmed using Rapid Diagnostic Test (RDT) through records at the local health facilities in the communities.

### Data collection process

Field research assistants visited selected households and sought at most two adults who have been diagnosed with malaria at the community health facility in the last one month. After study procedures had been explained to the respondents, informed consent obtained from them. RDT was performed on consenting respondents to confirm those who currently had malaria. Controls were also enrolled from the same area as those who had malaria. Selected cases were matched with controls with reference to age and sex.

A-semi-structured questionnaire was designed and used for interview. Section A of the questionnaire elicited information on sociodemographic characteristics while section B focused on challenges and possible factors limiting compliance with known and effective use of malaria treatment and preventive measures. The validity of the questionnaire was established through face and content validity criteria. The questionnaire was given to three experts in the field of public health. Each item on the instrument was examined for content, clarity, scope and relevance to the scope of the study. Data were collected, and the field research assistants were trained to ensure reliability of the information gathered.

### Data analysis

Data analysis was done using the Statistical Package for Social Science (SPSS) version 25. Data was analyzed using descriptive statistics presented with bar charts and pie charts. Likelihood ratio chi-square test was used to test the association between occurrence of malaria and likely factors that promote compliance/non-compliance to effective prevention and treatment measure while Fisher Exact test was used to compute odds ratio for association of LLIN use (Year-round) with predisposing factors for use/non-use. Level of significance was set at p = 0.05.

Ethical consideration: Ethical approval for the study was granted by the Health Research Ethics Committee of the State Ministry of Health (SMoH) of Western Lakes State, South Sudan (Reference Number: MOH/WLS/25/08/2017). The research was carried out in accordance with the principles of the Helsinki Declaration.

## Results

### Respondents’ socio-demographic characteristics

A total of 396 participants (198 cases, 198 controls) were interviewed. Table 1 shows the socio-demographic characteristics of the participants. The majority of the participants were aged 21-40 years (cases = 70.7%, controls = 73.7%). Higher proportion of the respondents were males (cases = 58%, controls = 60.6%) and most of them (cases = 85.9%, controls = 87.4%) were married. About 50% of those who tested positive for malaria did not have any formal education, whereas primary (7–8) qualification holders (44.9%) dominated the control group. Results further showed that the most predominant occupation among those who tested positive to malaria was farming/agricultural business while the white-collar job category was more prevalent among the controls.

**Table 1:** Socio-demographic characteristics of respondents (n = 396)

Variable	Cases (n = 198)	Controls (n = 198)
	Frequency (%)	Frequency (%)
**Age (years)**		
** 20 and below**	44(22.2)	32(16.2)
** 21–30**	88(44.4)	101(51.0)
** 31–40**	52(26.3)	45(22.7)
** 41–50**	11(5.6)	16(8.1)
**Above 50**	3(1.5)	4(2.0)
**Sex**		
** Female**	83(41.9)	78(39.4)
** Male**	115(58.1)	120(60.6)
**Marital Status**		
** Married**	170(85.9)	173(87.4)
** Single**	27(13.6)	21(10.6)
** Widow**	1(0.5)	4(2.0)
**Education**		
** No formal Education**	117(59.1)	23(11.6)
** Primary 1–6**	58(29.3)	43(21.7)
** Primary 7–8**	17(8.6)	89(44.9)
** Senior School**	5(2.5)	36(18.2)
** Tertiary**	1(0.5)	7(3.5)
**Occupation**		
** Artisan/Craftsmen/Traders**	53(26.8)	45(22.7)
** Farmer/Agric Business**	73(36.9)	67(33.8)
** Not working/Housewife**	17(8.6)	12(6.1)
** White Collar**	55(27.8)	74(37.4)

### Barriers and facilitators of compliance with preventive and treatment measures for malaria

Associations between occurrence of malaria and factors that could limit or promote compliance with preventive and treatment measures for malaria are presented in Table 2. Significant associations (p = 0.05) were observed between occurrence of malaria and identified factors like access to health facilities, satisfaction with healthcare services, access to LLIN, awareness of LLIN benefits, being taught on LLIN use, understanding of LLIN use and knowing that having bush near household promote occurrence of malaria. Two hundred and ninety-three respondents (74%) reported having fair access to health facilities while 372(93.9%) of them were at least satisfied with available healthcare services. Just above two-third (67.7%) of the respondents reported having access to LLIN, more than two-third of the respondents indicated being aware of the benefits of LLIN (91.4%), having been taught on LLIN use (64%) and understanding of how to use LLIN (61.6%). One hundred and seventy-two (43.4%) participants reported knowing that having bush near household promote occurrence of malaria.

**Table 2:** Association between occurrence of malaria and identified factors promoting non-compliance to effective prevention and treatment measures

	Occurrence of malaria				
Variable	No N (%)	Yes N (%)	□2	df	p-value
**Access to health facility**			25.453	4	= 0.001*
***Fair access***	165/293(56.3)	128/293(43.7)			
***Poor access***	33/103(32.0)	70/103(68.0)			
**Satisfaction with health care**			47.379	4	= 0.001*
***Very satisfied***	76/294(59.9)	118/294(40.1)			
***Satisfied***	18/78(23.1)	60/78(76.9)			
***Not satisfied***	4/24(0.17)	20/24(83.3)			
**Access to LLIN**			95.375	2	= 0.001*
***Yes***	179/268(66.8)	89/268(33.2)			
***No***	19/128(14.8)	109/128(85.2)			
**Awareness of LLIN benefit**			28.838	6	= 0.001*
***Yes***	195/362(53.9)	167/362 (46.1)			
***No***	3/34(8.8)	31/34(91.2)			
**Taught on LLIN use**			34.296	2	= 0.001*
***Yes***	52/256(59.4)	104/256(40.6)			
***No***	46/140(32.9)	94/140(67.1)			
**Understand LLIN use**			86.723	10	= 0.001*
***Yes***	156/244 (63.9)	88/244 (36.1)			
***Not very well***	35/94 (37.2)	59/94 (62.8)			
***No***	7/58 (12.1)	51/58 (87.9)			
**Knows bush near household promote occurrence of malaria**			31.636	2	= 0.001*
**Yes***Yes*	113/172(65.7)	59/172(34.3)			
***No***	85/224(37.9)	139/224(62.1)			

### Reasons for non-compliance with known effective prevention and treatment measures and associations with occurrence of malaria

Table 3 shows the summary of participants’ reasons for non-compliance with known effective prevention and treatment measures and associations with occurrence of malaria. The results showed that most participants were not using LLIN regularly because they do not have it (30.3%) and forgetting to use it most of the time (11.9%). About 44% of the participants did not consider using door/window net necessary, 116(29.3%) reported not knowing that using door/window nets could reduce malaria while 85 (21.4%) did not use it due to lack of funds. Notable reasons reported for not using insecticides included non-availability (50.3%) in the market and being too expensive (43.4%). Participants also indicated that they did not use mosquito repellant cream because it is too expensive (9.8%) and not readily available (9.6%). The reasons for participants’ preference for drip/injection at the expense of antimalarial drugs was the belief that it is more effective (23.7%) and works faster (21.7%).

**Table 3:** Association between occurrence of malaria and identified reasons for non-compliance to effective prevention and treatment measure

	Occurrence of malaria				
Variable	No N (%)	Yes N (%)	□2	df	p-value
**Reasons for not using LLIN regularly**			173.791	8	= 0.001*
***You do not like it***	0/5(0)	5(100.0)			
***You do not have***	18/120(15.0)	102/120(85.0)			
***You forget most time to use it***	14/47(29.8)	33/47(70.2)			
***You don't feel comfortable inside it***	5/27(18.5)	22/27(81.5)			
***It is not culturally accepted (or a taboo) to make use of it***	0(0)	0(0)			
***Other/No reason***	1(100.0)	0(0)			
**Reasons for non-use of Door/window net**			36.402	6	= 0.001*
***We do not consider it necessary to have window or door net***	62/176(35.2)	114/176(64.8)			
***We have no sufficient fund to fix it***	58/85(68.2)	27/85(31.8)			
***We don't know could help reduce malaria disease burden or any of health benefit***	64/116(55.2)	52/116(44.8)			
***Other/No reason***	0(0)	1(100)			
**Reasons for non-use of insecticides**			35.017	8	= 0.001*
***Too expensive***	65/172(37.8)	107/172(62.2)			
***The household see it as poisonous***	0(0)	4 (100)			
***It is not always available to buy***	126/199(63.3)	73/199(36.7)			
***It affects household health negatively***	6/15(40.0)	9/15(60.0)			
***Other/No reason***	1/6(16.7)	5/6(83.3)			
**Reasons for preference of drip/injection**			76.100	38	= 0.001*
***It works faster***	31/94(33.0)	63/94(67.0)			
***It is more effective***	38/86(44.2)	48/86(55.9)			
***I just like it***	10/21(47.6)	11/2(52.4)			
***No reason***	16/29(55.2)	13/29(44.8)			
**Reasons for non-use of mosquito repellant cream**			14.656	8	0.066
***Too expensive***	13/39(33.3)	26/39(66.7)			
***Not always available***	16/38(42.1)	22/38(57.9)			
***It affects household health negatively***	0(0)	1(100)			
***Others/No reason***	0(0)	3(100)			

* denotes significance at p = 0.05

**Figure 1:**
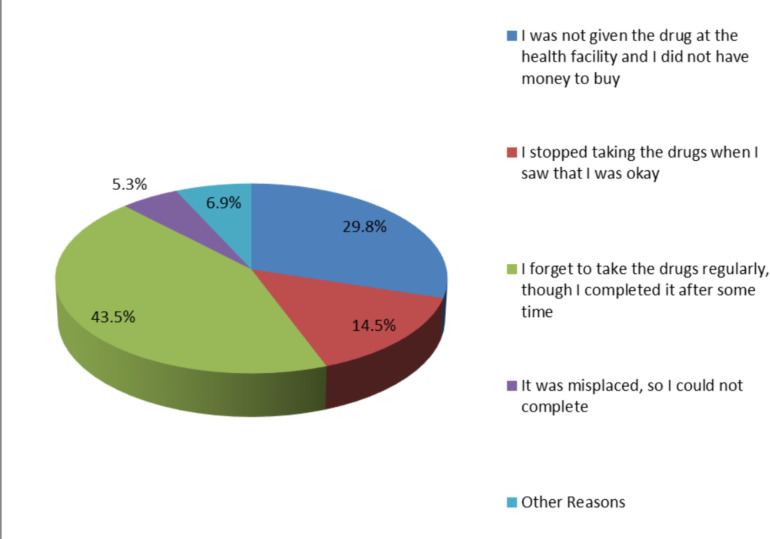
Reasons for non-compliance to taking and/or completing prescribed drugs

Figure 1 shows reasons for non-compliance to taking and/or completing prescribed antimalarial drugs among the respondents. Findings showed that 43.5% of the participants reported history of delayed or irregular consumption of prescribed drugs following last episode of malaria sickness though they completed the drug consumption. Fifty-six (14.5%) respondents reported they did not complete the drug intake as they stopped when they felt relief of symptoms.

### Factors influencing the year-round utilization of LLIN

Analysis of Odd Ratios (OR) for factors that influenced the year-round utilization of LLIN is presented in Table 4. Participants who had LLIN were 5.8 times more likely to use it regularly throughout the year than those who do not have it (OR: 5.78, 95% CI: 3.46 – 9.00). Those who were aware of the benefits of longlasting insecticide nets were 8.8 times more likely to use it year-round regularly (OR: 8.76, 95% CI: 3.02 – 25.37). Those who were taught on the use of LLIN were 3.4 times more likely to use LLIN year-round than those who had it but not taught (OR: 3.35, 95% CI: 2.17 – 5.18). It was also observed that those who understand the use of LLIN were 3.8 times more likely to use it regularly year-round compared to those who do not understand its use (OR: 3.80, 95% CI: 2.01 – 7.20).

**Table 4:** Factors influencing year-round use of LLIN

Variables	Use LLIN regularly/Year round Frequency (%)	Don't use regularly /Year-round Frequency (%)	□^2^	OR (95% CI)	p-value
**Have LLIN**			54.40	5.78(3.46 – 9.00)	= 0.001*
**Yes**	169/268 (63.1)	99/268(36.9)			
**No**	30/128 (23.4)	98/128(76.5)			
**Aware of benefit**			22.04	8.76(3.02 – 25.37)	= 0.001*
**Yes**	195/362 (53.9)	167/362(46.1)			
**No**	4/34 (11.8)	30/34(88.2)			
**Taught on LLIN use**			30.67	3.35(2.17 – 5.18)	= 0.001*
**Yes**	155/256(60.5)	101/256(39.4)			
**No**	44/140(31.4)	96/140(68.6)
Understand LLIN use			18.54	3.80(2.01 – 7.20)	= 0.001*
**Yes**	185/338(54.7)	153/338(45.3)			
**No**	14/58(24.1)	44/58(75.9)			

## Discussion

This study aimed to determine the challenges and factors that limit compliance with the utilization of known and effective malaria treatment and preventive measures in Wulu, South Sudan. Consequently, the findings are pertinent to better understanding of factors limiting the achievement of the set malaria control programme objectives and also provide opportunity for identifying areas that require more attention in the malaria control initiative.

The finding of cost (lack/inadequacy of fund) as a factor for poor compliance suggests the possible role of poverty and perhaps population socio-economic status in the control effort to reduce malaria and its burden. This is consistent with the findings of Sintasath et al

(18) and Ayele et al (19). It is a practice as part of the malaria control programme in South Sudan to distribute LLINs freely to the targeted population of mothers and under five children. Many individuals and families could not afford to self-purchased LLINs, repellant creams, indoor residual spray, door and window barriers etc, whenever they could not get it free largely due to lack or inadequate fund.

Inadequate access to preventive and control measures was found to be a limiting chance of compliance. Although about 57% reported having had access to LLIN, access to other measures such as mosquito repellant creams, door and window barriers, indoor residual spray etc were found to be inadequate. These factors were found to have significant association with occurrence of malaria. Few proportions of the population, however, reported unavailability of these preventive and control measures even when they have resource to procure. Delay or poor timing in the distribution (free) and low retention of long-lasting insecticide nets beyond raining season for future/continuous use by the participants needed to be looked into to improve utilization and resource management.

The poor compliance to malaria control measures by individual and households could be a factor contributing to the perennial transmission of malaria in Wulu and perhaps other regions of the country, despite seasonal rainfall pattern, with long breaks. The study of Musoke et al (20) showed that as the households continued to use many of the malaria prevention methods in the integrated approach they really benefited as revealed through observed reduction in mosquitoes indoors and malaria occurrence. Malaria prevention methods in the integrated approach included sleeping under long-lasting insecticidal nets, screening in windows and ventilators, remove mosquito breeding sites, and ensure closing of doors early in the evening (20). Similar finding was reported by Pinchoff et al (6) which studied individual and Household Level Risk Factors Associated with Malaria in Nchelenge District, Northern Zambia. Factors of compliance to preventive and control measures have to be continuously monitored, with findings addressed if the objectives are to be promptly achieved across populations.

Factors such as being educated, possessing understanding and good awareness of the preventive and control measures were associated with occurrence of malaria. Promoting the practice and monitoring the practice could help improve compliance to utilization and invariably reduce the incidence of malaria. A Kenyan intervention study that investigated the proportion of ITNs used the prior night in households which had received ITNs, hanging supplies, and education reported that 30% of ITNs distributed were not used the prior night. Heat was reported as the main reason for non-use (21). Zuradam (22) also found that those who had good knowledge of the importance of ITN utilize it more. It was further explained that people who realize the importance of LLIN would probably go to purchase it while those that received free nets might not consider it needful, hence the refusal to use it. This therefore suggests the need for much education regarding the importance of LLINs before embarking on free LLINs distribution exercise. Previous studies (23–26) on various health service research projects globally recommended the need to understand how local knowledge, belief and practice might influence the effectiveness of target interventions in control programmes.

Having LLIN, being aware of LLIN, being taught about the use of LLIN and understanding about the use of LLIN were significantly associated (all at p = 0.001) with and increased odds for its use throughout the year. Sagare et al

(27) found that owning more than one bed net was slightly associated with always sleeping under an ITN during pregnancy (RR: 1.13; 95%

CI: 1.00, 1.2) among Ugandan pregnant women. Nganda et al (28) similarly found that knowledge of malaria was significantly associated with net usage. They further observed that respondents with high knowledge of malaria were 2 times more likely to use nets compared to those with low scores. Sangare et al (27) did not find a significant association between knowledge of malaria and bed net. This might be explained by the difference of respondents’ characteristics. The present study enrolled both genders as well as married and unmarried individuals while Sangare et al’s (27) respondents were pregnant women only. Increasing awareness on the importance of LLINs needs to be intensified in communities to improve usage and consequently reduce malaria occurrence. Sangare et al (27) further reported that women who always slept under an ITN during pregnancy were more likely to be influenced by radio/poster advertisements than being given an ITN free of charge (RR: 1.48; 95% CI: 1.24, 1.76).

Preference for injection appears prevalent among respondents diagnosed with malaria and is significantly associated with malaria occurrence. Poor understanding or perception in the use of injection by the population could create room for wrong implementation of guideline in the treatment of malaria. South Sudan like many developing countries have weak health system with inadequate control or monitoring of health facilities especially those within communities and not owned by government. Any kind of malaria treatment could be permitted by private community clinics, based on the perception and request of the clients and not according to the country’s malaria treatment guideline. Local health authorities and the health system needed to be well strengthened and supported in order to effectively monitor the kind and quality of care being delivered to the population. This will limit infection spread and help in reducing resistance to treatment medications. About 44% of the respondents reported forgetting to take malaria drugs regularly, and this should be a concern to the clinicians. It is important for clinicians to develop methods or strategy that will ensure that clients take and complete medications as prescribed. Involvement of other members of households in the taking of drugs could be beneficial to address some of the reasons that were given by the participants.

The level of illiteracy in most parts of South Sudan is still very high, the country’s 2018 literacy rate for population group aged 15 years or older as reported by UNESCO was 34.52% (29). This is evidenced by the finding that only 6 respondents of this study had tertiary education. The educational status of individuals or care givers is beneficial to the enhancing of utilization and reduction of malaria burden. Girum et al (30) found that educated individuals were more likely to practice malaria prevention practice than illiterates (AOR = 2.6, (95% CI: 1.8–3.6). The reports of other related studies (31,32) also showed that associations exist between compliance to preventive measures and level of education. Programmes aiming to improve both maternal health and maternal education may reduce the incidence of severe malaria in children and should therefore be more advocated in Wulu and in areas with similar epidemiological patterns for malaria. More should also be done in the area of improving access to quality education.

Respondents’ recall bias is a factor that could threaten the internal validity of this study. Randomization in sampling and the use of pictorial materials with explanations were done to minimize its effect. Researchers also ensured that participants have lived in the locality for at least 6 months prior to the study.

Notable reasons for non-compliance with available treatment, control and preventive measures include: not having LLIN, forgetfulness regarding the use of LLIN and prescribed drugs, preference for injection, not considering having door/window barriers necessary. In addition, the study showed that respondents’ having LLIN, being aware of its benefits, being taught on its use as well as having understanding of its use were more likely to make a year-round utilization of LLIN. Hence, poor access to and knowledge of malaria treatment and preventive measures are major barriers to their effective utilization in Wulu.

Factors identified and recommendation made would be of great benefit to achieving the control objectives if well looked into and implemented by health policy makers, health partners and government authorities. Since pregnant women and young children bear the greatest burden and target of most control programmes, future studies of this nature should be structured to target these two groups.

## References

[1] WHO (2017). Malaria: Key facts. https://www.who.int/news-room/factsheets/detail/malaria.

[2] WHO World malaria report 2016. https://apps.who.int/iris/bitstream/handle/10665/252038/9789241511711-eng.pdf.

[3] WHO World malaria report 2018. https://www.who.int/malaria/publications/world-malaria-report-2018/en/.

[4] WHO (2020). South Sudan National Malaria Strategic Plan, 2014 – 2021, launched. https://www.afro.who.int/news/south-sudannational-malaria-strategic-plan-2014-2021launched.

[5] WHO (2015). Global technical strategy for malaria 2016–2030.

[6] Bhatt, S, Weiss DJ, Cameron E, Bisanzio D, Mappin B, Dalrymple U (2015). The effect of malaria control on Plasmodium falciparum in Africa between 2000 and 2015. Nature.

[7] Bhatt S, Weiss DJ, Gethinget PW (2015). Coverage and system efficiencies of insecticide-treated nets in Africa from 2000 to 2017. Elife.

[8] Meremikwu, MM, Donegan S, Sinclair D, Esu E, Oringanje C (2012). Intermittent preventive treatment for malaria in children living in areas with seasonal transmission. Cochrane Database Syst Rev.

[9] Poirot E, Skarbinski J, Sinclair D, Kachur SP, Slutsker L, Hwang J (2013). (2013): Mass drug administration for malaria. Cochrane Database Syst Rev.

[10] Eisele TP, Larsen DA, Anglewicz PA, Keating J, Yukich J, Bennett A (2012). Malaria prevention in pregnancy, birthweight, and neonatal mortality: a metaanalysis of 32 national cross-sectional datasets in Africa. Lancet Infect Dis.

[11] Pinchoff J, Chaponda M, Shields TM, Sichivula J, Muleba M, Mulenga M, Kobayashi T, Curriero FC, Moss WJ (2016). Individual and Household Level Risk Factors Associated with Malaria in Nchelenge District, a Region with Perennial Transmission: A Serial Cross-Sectional Study from 2012 to 2015; Southern Africa International Centers of Excellence for Malaria Research. PLoS ONE.

[12] Flaxman AD, Fullman N, Otten MW, Menon M, Cibulskis RE, Ng M (2010). Rapid scaling up of insecticide-treated bed net coverage in Africa and its relationship with development assistance for health: a systematic synthesis of supply, distribution, and household survey data. PLoS Med.

[13] Chanda E, Remijo CD, Pasquale H, Babab SP, Lakob RL (2014). Scale-up of a programme for malaria vector control using long-lasting insecticide-treated nets: lessons from South Sudan. Bull World Health Organ.

[14] WHO (2020). South Sudan sets to improve malaria surveillance through a sentinel site strengthening approach. https://www.afro.who.int/pt/node/10404.

[15] MOH, South Sudan (2008). Strategy for increasing coverage of long-lasting insecticide treated mosquito nets (LLINs) in South Sudan.

[16] United Children Emergency Fund (UNICEF) (2004). Malaria: A major cause of child death and poverty in Africa. https://www.unicef.org/publications/index_19019.html.

[17] Charan J. Biswas T. (2013). (2013) How to Calculate Sample Size for Different Study Designs in Medical Research. Indian J Psychol Med.

[18] Sintasath DM, Ghebremeskel T, Lynch M (2005). (2005): Malaria prevalence and associated risk factors in Eritrea. Am J Trop Med Hyg.

[19] Ayele DG, Zewotir TT, Mwambi HG (2012). Prevalence and risk factors of malaria in Ethiopia. Malar J.

[20] Musoke D, Karani G, Ndejjo R, Okui P, Boses M (2016). Experiences of households using integrated malaria prevention in two rural communities in Wakiso district, Uganda: a qualitative study. Malar J.

[21] Alaii JA, Hawley WA, Kolczak MS, ter Kuile FO, Gimnig JE, Vulule JM, Odhacha A, Oloo AJ, Nahlen BL, Phillips-Howard PA (2003). Factors affecting use of permethrin-treated bed nets during a randomized controlled trial in western Kenya. Am J Trop Med Hyg.

[22] Zuradam SF Factors associated with use and non-use of mosquito nets for children less than 5 years of age in the Mfantseman municipality, Ghana. http://epublications.uef.fi/pub/urn_nbn_fi_uef-20121116/urn_nbn_fi_uef-20121116.pdf.

[23] Adongo PB, Kirkwood B, Kendall C (2005). How local community knowledge about malaria affects insecticide-treated net use in northern Ghana. Trop Med Int Health.

[24] Wongsrichanalai C, Barcus MJ, Muth S, Sutamihardja A, Wernsdorfer WH (2007). A Review of Malaria Diagnostic Tools: Microscopy and Rapid Diagnostic Test (RDT). Am J Trop Med Hyg.

[25] Ajala AS, Wilson NA (2013). Local Aetiology and Pathways to Care in Malaria among the Ibibio of South-coastal Nigeria. Health, Culture and Society.

[26] Deressa W, Yihdego YY, Kebede Z, Batisso E, Tekalegne A, Dagne GA (2014). Effect of combining mosquito repellent and insecticide treated net on malaria prevalence in Southern Ethiopia: a cluster-randomised trial. Parasit Vectors.

[27] Sangaré LR, Weiss NS, Brentlinger PE, Richardson BA, Staedke SG, Kiwuwa MS (2012). Determinants of Use of Insecticide Treated Nets for the Prevention of Malaria in Pregnancy: Jinja, Uganda. PLoS ONE.

[28] Nganda RY, Drakeley C, Reyburn H, Marchant T (2004). Knowledge of malaria influences the use of insecticide treated nets but not intermittent presumptive treatment by pregnant women in Tanzania. Malar J.

[29] United Nations Educational, Scientific and Cultural Organization (UNESCO) (2020). South Sudan -Sustainable Development Goals: Education and Literacy. http://uis.unesco.org/en/country/ss.

[30] Girum T, Hailemikael G, Wondimu A (2017). Factors affecting prevention and control of malaria among endemic areas of Gurage zone: an implication for malaria elimination in South Ethiopia, 2017. Tropical diseases, Travel Medicine and Vaccines.

[31] Roberts D, Matthews G (2016). Risk factors of malaria in children under the age of five years old in Uganda. Malar J.

[32] Snyman K, Mwangwa F, Bigira V, Kapisi J, Clark TD (2015). Poor housing construction associated with increased malaria incidence in a cohort of young Ugandan children. Am J Trop Med Hyg.

